# SPA-STOCSY: an automated tool for identifying annotated and non-annotated metabolites in high-throughput NMR spectra

**DOI:** 10.1093/bioinformatics/btad593

**Published:** 2023-10-04

**Authors:** Xu Han, Wanli Wang, Li-Hua Ma, Ismael AI-Ramahi, Juan Botas, Kevin MacKenzie, Genevera I Allen, Damian W Young, Zhandong Liu, Mirjana Maletic-Savatic

**Affiliations:** Jan and Dan Duncan Neurological Research Institute at Texas Children’s Hospital, Houston, TX 77030, United States; Department of Pediatrics-Neurology, Baylor College of Medicine, Houston, TX 77030, United States; Jan and Dan Duncan Neurological Research Institute at Texas Children’s Hospital, Houston, TX 77030, United States; Graduate Program of Quantitative & Computational Biosciences, Baylor College of Medicine, Houston, TX 77030, United States; Advanced Technology Cores, Baylor College of Medicine, Houston, TX 77030, United States; Jan and Dan Duncan Neurological Research Institute at Texas Children’s Hospital, Houston, TX 77030, United States; Department of Molecular and Human Genetics, Baylor College of Medicine, Houston, TX 77030, United States; Jan and Dan Duncan Neurological Research Institute at Texas Children’s Hospital, Houston, TX 77030, United States; Department of Molecular and Human Genetics, Baylor College of Medicine, Houston, TX 77030, United States; Advanced Technology Cores, Baylor College of Medicine, Houston, TX 77030, United States; Center for Drug Discovery, Baylor College of Medicine, Houston, TX 77030, United States; Jan and Dan Duncan Neurological Research Institute at Texas Children’s Hospital, Houston, TX 77030, United States; Department of Electrical and Computer Engineering, Statistics, and Computer Science, Rice University, Houston, TX 77005-1827, United States; Jan and Dan Duncan Neurological Research Institute at Texas Children’s Hospital, Houston, TX 77030, United States; Center for Drug Discovery, Baylor College of Medicine, Houston, TX 77030, United States; Jan and Dan Duncan Neurological Research Institute at Texas Children’s Hospital, Houston, TX 77030, United States; Department of Pediatrics-Neurology, Baylor College of Medicine, Houston, TX 77030, United States; Jan and Dan Duncan Neurological Research Institute at Texas Children’s Hospital, Houston, TX 77030, United States; Department of Pediatrics-Neurology, Baylor College of Medicine, Houston, TX 77030, United States

## Abstract

**Motivation:**

Nuclear magnetic resonance spectroscopy (NMR) is widely used to analyze metabolites in biological samples, but the analysis requires specific expertise, it is time-consuming, and can be inaccurate. Here, we present a powerful automate tool, SPatial clustering Algorithm-Statistical TOtal Correlation SpectroscopY (SPA-STOCSY), which overcomes challenges faced when analyzing NMR data and identifies metabolites in a sample with high accuracy.

**Results:**

As a data-driven method, SPA-STOCSY estimates all parameters from the input dataset. It first investigates the covariance pattern among datapoints and then calculates the optimal threshold with which to cluster datapoints belonging to the same structural unit, i.e. the metabolite. Generated clusters are then automatically linked to a metabolite library to identify candidates. To assess SPA-STOCSY’s efficiency and accuracy, we applied it to synthesized spectra and spectra acquired on *Drosophila melanogaster* tissue and human embryonic stem cells. In the synthesized spectra, SPA outperformed Statistical Recoupling of Variables (SRV), an existing method for clustering spectral peaks, by capturing a higher percentage of the signal regions and the close-to-zero noise regions. In the biological data, SPA-STOCSY performed comparably to the operator-based Chenomx analysis while avoiding operator bias, and it required <7 min of total computation time. Overall, SPA-STOCSY is a fast, accurate, and unbiased tool for untargeted analysis of metabolites in the NMR spectra. It may thus accelerate the use of NMR for scientific discoveries, medical diagnostics, and patient-specific decision making.

**Availability and implementation:**

The codes of SPA-STOCSY are available at https://github.com/LiuzLab/SPA-STOCSY.

## 1 Introduction

Many of the diseases that plague developed societies—obesity, diabetes, cardiovascular and neuro-degenerative diseases, cancer—are caused by or associated with faulty metabolic regulation. To measure composite metabolic changes, the medical community is increasingly relying on metabolomics (the study of all metabolites in a given sample) for quantitative phenotyping and biomarker discovery (Maletić-Savatić *et al.* 2008, Vingara *et al.* 2013, Botas *et al.* 2015, Zhu *et al.* 2015, Markley *et al.* 2017, Newgard 2017, Kennedy *et al.* 2018, Emwas *et al.* 2019). However, metabolomics is not yet fully accepted as a critical tool for clinical assessments and mechanistic discoveries, mostly due to the absence of standardized protocols, reproducibility of results, and complex data analysis. These issues are being addressed by the development of standardized operating procedures, improving data quality control, and enhancing analytical tools. The two main platforms used to acquire metabolomic data are mass spectrometry (MS) and nuclear magnetic resonance spectroscopy (NMR). MS is more sensitive than NMR; by detecting more metabolites, MS can survey a large fraction of the metabolome. However, it is intrinsically qualitative. NMR is rapid, nondestructive, and noninvasive, and it requires minimal sample preparation. Most importantly, it is quantitative (NMR signal intensity is proportional to sample concentration) at a dynamic range of 2 × 10^5^ and very reproducible (coefficient of variation, CV 1–2%), making it ideal for on-site diagnostics and biomarker discovery (de Graaf 2007). Because small changes in some metabolites, such as blood cholesterol and glucose, have serious health implications, identifying subtle but significant metabolic changes at the level of the whole metabolome can have a substantial clinical impact. Amassing relevant quantitative and highly reproducible data, such as those provided by the NMR, could transform biology and medicine.

Identifying signals in NMR spectra may seem straightforward, but there are several practical challenges in decomposing NMR spectra to eventually derive the metabolome content (de Graaf 2007). First, NMR data analysis is operator-based and time-consuming due to the complex nature of the data: the spectra contain thousands of resonances that may belong to hundreds of metabolites and although each metabolite has a unique spectral pattern, peaks of different metabolites may overlap. Second, sample (pH, temperature, ionic strength, etc.) and operation-related instrument issues can affect line shapes of signals, causing them to differ from the reference library’s ideal signals; this makes matching the spectra with the library difficult. Third, a real sample may contain components not found in the library, such as a breakdown product of an uncommon food or a rare drug (de Graaf 2007, MacKenzie *et al.* 2020). Thus, NMR spectra may not correspond precisely to the weighted sums of ideal reference library components. Analyzing NMR spectra is also difficult due to their high dimensionality: thousands of variables (datapoints) correspond to thousands of dimensions, which can be computationally intense. Yet, NMR signal analysis methods should not attempt to simplify these computations by making assumptions about metabolites’ number and identity, as it is critical to extract the actual chemical, structural, and metabolic pathway information. For all these reasons, NMR-based metabolomics, despite its intrinsic quantitative and highly reproducible nature, is still rarely used for metabolomics discoveries.

To help surmount these challenges, several computational tools have been developed to perform standard data preprocessing and obtain phased, baseline-corrected, chemical shift-referenced, and normalized spectra (van Beek 2007, Günther *et al.* 2000, Ludwig and Günther 2011, Worley and Powers 2014, Pang *et al.* 2021). Existing computational and statistical tools for metabolomic profiling can then be applied to the processed spectra to extract the biologically useful information (Hollywood *et al.* 2006, Lewis *et al.* 2009, Xia *et al.* 2009, Allen and Maletić-Savatić 2011, Zheng *et al.* 2011, Hao *et al.* 2012, Allen *et al.* 2013, Peterson *et al.* 2013, Zhang *et al.* 2015). However, these methods, led by Statistical Total Correlation Spectroscopy (STOCSY) (Cloarec *et al.* 2005) and its variations (Robinette *et al.* 2013) have yet to accurately identify metabolites from the NMR spectra because they do not successfully solve the high dimensionality of the covariance matrix estimation and the overlapping signals. In a typical NMR spectrum, the number of chemical shift intervals (ppm values) can range from 3000 to 8000 and the number of samples can range from several to hundreds. Signals belonging to a single metabolite can be close to each other but also remote. In addition, adjacent points on a spectrum often demonstrate high correlation, which makes it hard to estimate distal dependence. To solve some of these problems, some methods first group NMR signals in a certain way before using STOCSY: e.g. Cluster Analysis Statistical Spectroscopy (CLASSY) uses a correlation matrix of peaks and an intersection matrix to determine local clusters and explore the intra-metabolite connections (Robinette *et al.* 2009). Consequently, it identifies the inter-metabolite connections by hierarchical clustering of those local clusters. Another approach, Statistical Recoupling of Variables (SRV), groups variables by scanning the covariance/correlation ratio landscape of consecutive variables. It then combines the grouped variables into super-clusters that represent variables with similar physical, chemical, and biological properties (Blaise *et al.* 2009). STOCSY is then applied to the super-clusters to help identify the metabolites. There have also been attempts to extend STOCSY to accelerate the metabolite identification process. For example, the STOCSY-scaling method scales down the contribution of the metabolites with dominant intensities, such as glucose, which could explain most of the variation between the biological groups (Maher *et al.* 2012). Such scaling makes it possible to explore and identify metabolites of diagnostic interest covered by those dominant metabolites. Subset Optimization by Reference Matching (STORM), on the other hand, was developed to advance biomarker discovery by applying STOCSY on selected subsets of spectra that contain specific spectroscopic signatures that distinguish between different human populations (Posma *et al.* 2012). More recently, Resolution-Enhanced STORM (RED-STORM) was developed as an extension of STORM in a probabilistic framework (Posma *et al.* 2017). Finally, Peak Overlap Detection by Clustering Analysis and Sorting of Traces (POD-CAST), which is applied to NMR data after STOCSY, aims to overcome peak overlap issues and provides a better peak list for database queries (Hoijemberg and Pelczer 2018). Despite all these efforts, however, metabolite identification remains time-consuming and requires the operator to have highly specialized knowledge and perform complex procedures, while still failing to identify novel metabolites in the spectra.

**Figure 1.**
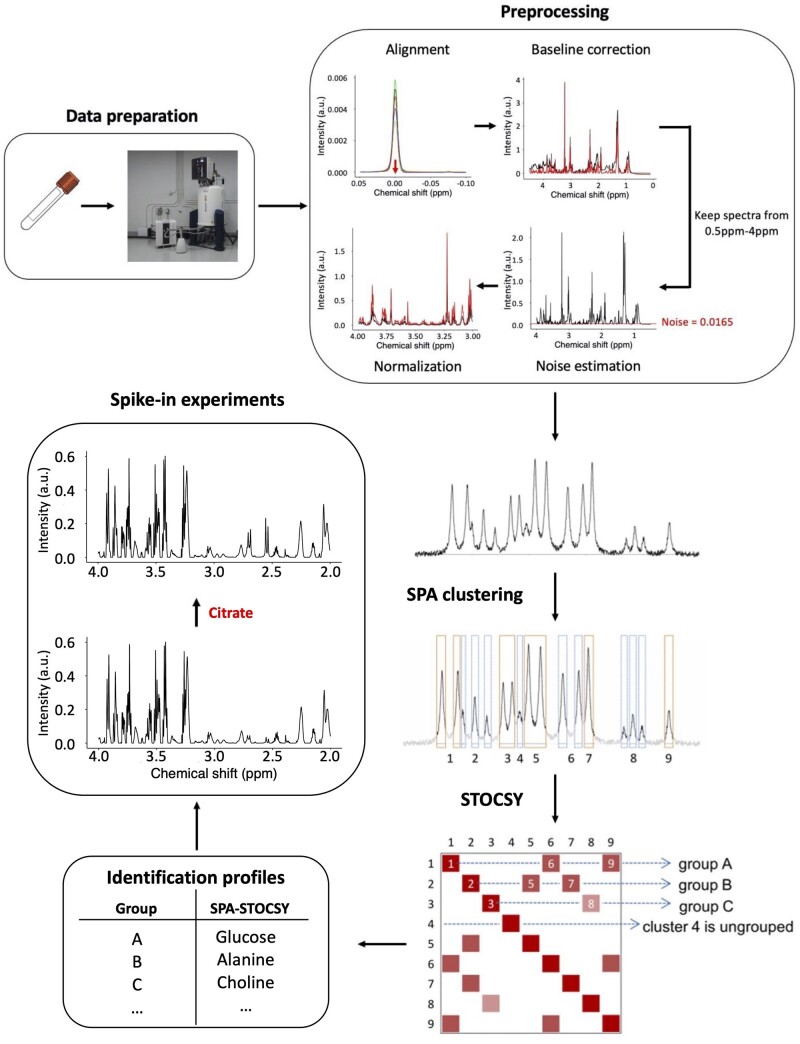
SPA-STOCSY flow chart. A set of spectra are experimentally acquired by NMR and preprocessed. The mean spectrum of preprocessed spectra is shown before and after the SPA clustering. SPA automatically identifies clusters (outlined in orange or blue) that correlate strongly across multiple spectra (*N* > 8). STOCSY of these clusters then automatically generates groups of clusters predicted to be from the same metabolite, i.e. the ^1^H chemical shifts of the clusters in each SPA-STOCSY group are predicted to be most likely from the same metabolite. Using a reference library, SPA-STOCSY generates an identification profile for each metabolite by summarizing the information from each cluster. Finally, to test the authenticity of identified metabolites, spike-in experiments can be performed. SPA, SPatial clustering Algorithm; STOCSY, Statistical Total Correlation Spectroscopy.

To address this gap and accelerate the application of NMR metabolomics in medicine and biology, we present a novel, automated approach that identifies metabolites with spectral patterns in a known database (reference library) and provides insights into those not in the reference library. We took advantage of two existing algorithms: Spatial clustering algorithm (SPA) and STOCSY (Cloarec *et al.* 2005) (Fig. 1). SPA exploits strong correlations among datapoints from the multiplets (multiple peaks belonging to the same metabolite) and identifies local spatial clusters of contiguous datapoints highly likely to arise from the same metabolite cluster. With the SPA-derived clusters as input, STOCSY is then used to highlight the correlation map and arrange clusters into highly correlated groups containing signals from the same metabolite. Once combined, the analytical power of these two algorithms synergistically increases and, compared to the existing methods, enables automatic, accurate, and fast identification of metabolites in the spectra.

## 2 Materials and methods

### 2.1 NMR simulation model

We generated spectral intensities as


(1) $$y_{i}\left(f\right)=S_{i}\left(f\right)+N_{i}\left(f\right),$$

where *f* denotes the datapoints in terms of ppm, *f* = 1, …, *p*, and *i* denotes the samples, *i* = 1, …,*n*. A typical simulated spectrum with 50 metabolites is shown. (Supplementary Fig. S1a). We specified the noise term *N*(*f*) as an autoregressive process of order 1, i.e. AR(1), in simulations we used ρ = 0.9 (Supplementary Fig. S1b). Our choice of error term seemed reasonable based on the characteristics of the real data, and it has also been adopted in simulation models for other high-throughput spectral data, such as mass spectrometry data (Cruz-Marcelo *et al.* 2008). Let *L* be the total number of metabolites present in a sample. *M*(*f*) is defined as reference spectra (scaled to have a maximum of 1) that the metabolite *l* resonates at the chemical shift, *f*, i.e. *M_l_*(*f*) ∈ [0, 1] (Supplementary Fig. S1c), and Σ ∈ *R*^*L*×*L*^ as a correlation matrix, denoting the correlation between the metabolites in the sample. The matrix *S*_(*n*×*p*)_ of population spectra, with elements *S*_*i*_(*f*). *S* is defined as


(2) $$S=\left(H\Sigma +1_{n}\alpha^{T}\right)M,$$

where *M* ∈ *R*^*L*×*p*^ has elements *M*_*I*_(*f*), *H* ∈ *R*^*n*×*L*^ has elements *H*_*i*_ ∈ ^*iid*^*N*(0, ∅^2^), denoting the variation of metabolite *I* in the *i-*th sample and α ∈ *R*^*L*×1^ such that α_l_ ∈ ^*iid*^*X*^2^(*γ*), denoting the mean concentration of each metabolite in the sample.

In simulations, we fixed *L*, the total number of metabolites in the samples, and *M*, the metabolite reference spectra. We then generated different datasets with different values of *n*, the sample size, *γ*, the degree of freedom for generating metabolite concentrations, and ϕ, the variance of generating metabolite variation. These parameters, in turn, determined the signal-to-noise ratio (SNR), calculated as the variance of signals divided by the variance of noise:


$$\mathrm{SNR}=\frac{2L\gamma +L\phi^{2}}{n}.$$

Parameter values were chosen to imitate experimental data from our lab, acquired on an 800 MHz NMR spectrometer (Bruker Corp.). In particular, the mean value to generate metabolite concentrations and the variance for simulation were determined based on summary statistics from data that included both *in vivo* and *in vitro* experiments, acquired from humans, mice, and fruit flies. Means and variances of the intensities at the 25th, 50th, and 75th percentiles were calculated from this experimental data. Simulation parameters were set to have summary statistics of the simulated data matching this range of values.

### 2.2 Simulation design

We designed three sets of synthesized spectra in which the total number of metabolites in the sample, *L*, was fixed at 10, 30, or 50 metabolites. The spectra were generated for the chemical shift range of 0.5–4.0 ppm because in the biological samples, this part of the spectra contains the most complex and dense data from relevant metabolites. Each reference spectrum was scaled to its maximum intensity so that each spectrum was within the range (0, 1). The scaled spectra were then used to construct the matrix *M* in the three simulated scenarios, according to the different values of *L*. We generated data according to models (1) and (2). In all simulations, we set Σ = *I*. We fixed *γ* = 60 and had either ∅ = 12 or 25. We used the partial autocorrelation function (PACF) to assess autocorrelation within the dataset and determine the appropriate lag value, denoted as *k*. The goal was to identify the lag at which the partial autocorrelation falls within the 95% confidence interval. This approach automatically suggests the optimal window size for a group of spectra. Within this window, neighboring datapoints exhibit stronger correlations and are more likely to originate from the same metabolite. We performed analysis on two sample sizes (*n* = 50, 100). Each dataset (*n* = 50 or 100 samples per dataset) had a different combination of concentrations of the reference metabolites. However, the mean intensity of each spectrum was always the same.

### 2.3 Spatial clustering algorithm (SPA)

**Figure 2.**
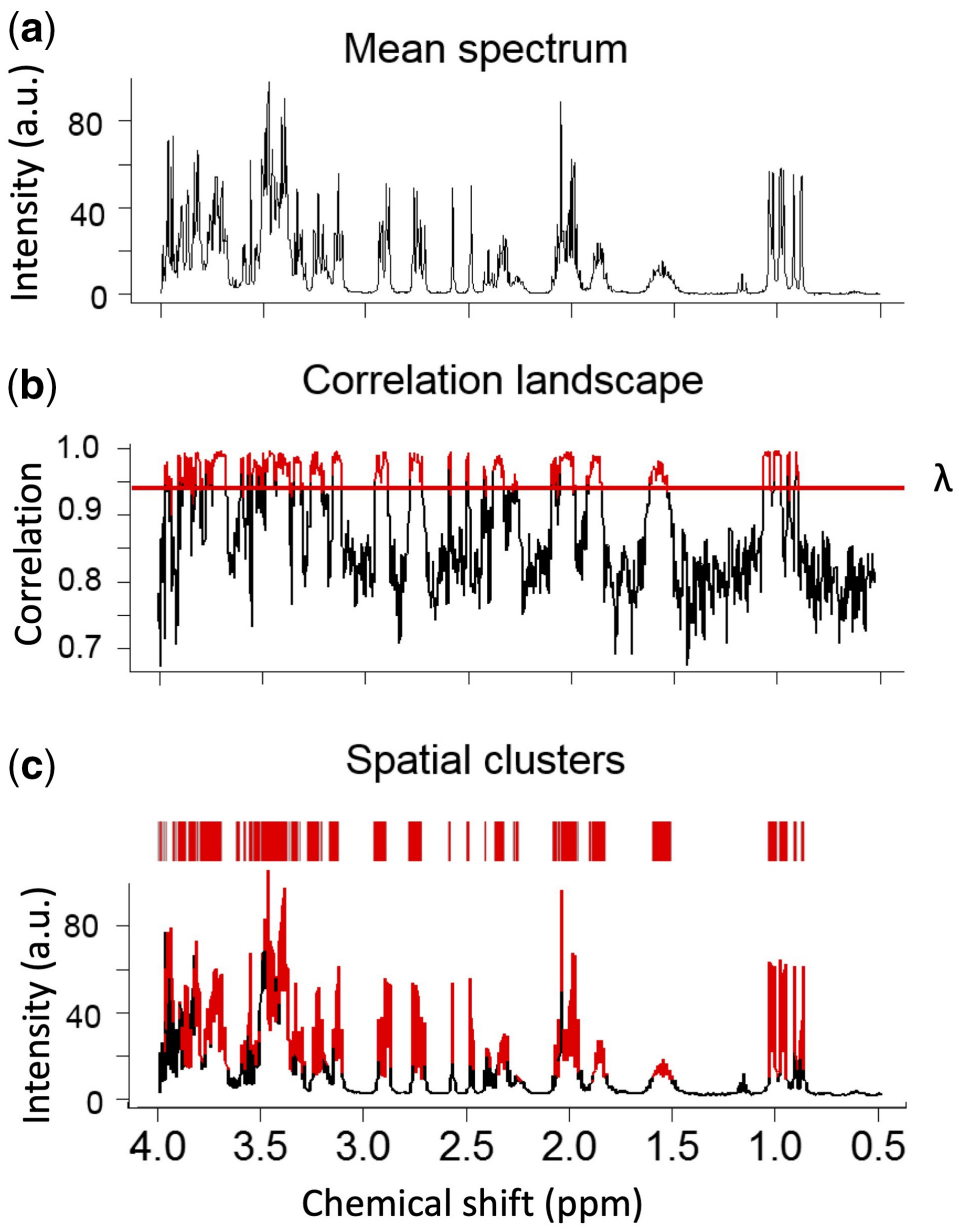
SPA flowchart. (a) The mean spectrum of the simulated NMR spectra of 10 metabolites. (b) Correlation landscape and the threshold *λ*. (c) Identified SPA clusters putatively belong to the same structural units of a metabolite. Red indicates clustered regions.

SPA first generates a correlation landscape, since variables from the same functional unit tend to have stronger correlations with each other than variables from different functional units or the baseline (Fig. 2a and b). To achieve an accurate record of the relationships among individual variables, we designed a moving average approach to scan the correlations among consecutive variables. This moving average calculates the correlation landscape as the average pair-wise Pearson correlation between adjacent variables in a window of fixed size. Let us define


(3) $$Q\left(j,\;j+k\right)=\;\frac{2}{k(k-1)}\sum_{j^{\prime }=1}^{k}\sum_{j^{\prime \prime }=j^{\prime }+1}^{k}\rho ({x.}_{j+j^{'}\;},\;{x.}_{j+\;j^{\prime \prime }}),$$

where *Q* is the correlation landscape, *j* is the index of the first variable in the window, and *k* is the window size. ρ(.) denotes the Pearson correlation coefficient. *X* denotes the dataset under study, whose columns represent the datapoints at each ppm value and rows represent sample.

Next, we improved the stability of the partial correlation landscape by kernel smoothing. Based on our simulated data, the Epanechnikov kernel,


(4) $$g\left(t\right)=\;\frac{3}{4}(1-t^{2})\;\mathrm{if}\;\left|t\right|\leq 1\;\mathrm{and}\;0\;\mathrm{otherwise},$$

and the tricube kernel,


(5) $$g\left(t\right)={\left(1-{\left|t\right|}^{3}\right)}^{3}\;\mathrm{if}\;\left|t\right|\leq 1\;\mathrm{and}\;0\;\mathrm{otherwise},$$

are best suited for the correlation landscape generated by the spectral data. The Epanechnikov and tricube kernels are kernel functions that give higher weights to the central point within a small window. In the case of NMR spectra, resolving the issue of metabolite overlap is a significant challenge. Singlet peaks representing specific metabolites are often obscured by signals from neighboring metabolites. By assigning higher weights to these central points, the Epanechnikov and tricube kernels effectively enhance the correlation for peaks affected by overlapping issues. In severe cases of overlapping, the tricube kernel, with its power of three, is superior to the Epanechnikov kernel.

To identify SPA clusters in a spectrum (Fig. 2c), we needed to set a threshold on the correlation landscape *λ*. Although this threshold can be empirically determined, we set it by optimizing the correlation thresholds that maximize the stability for ppm memberships between two equally split datasets out of *X* (the whole dataset under study). We designed an automated procedure based on prediction strength (Tibshirani and Walther 2005). It is calculated as the median proportion of correctly allocated variables across all spatial clusters in a sampled set (Tibshirani and Walther 2005). This procedure first randomly splits the data into two sets of equal sample size, set-1 and set-2. The goal is to find the threshold λ at which the spatial clusters become most stable between set-1 and set-2. At the same time, the grouped variables should be as informative as possible to represent the chemical structural information of the metabolites. To accomplish this goal, we constructed a *p* × *p* co-membership matrix *D*, representing how well the memberships in set-1 can predict the memberships in set-2. If variable *i* in the set-1 and variable *i*_0_ in the set-2 are in the same cluster, then *D*(*i*, *i*_0_) equals 1, and if not, it equals 0. Let *c* be the total number of clusters, *m*_1_,…,*m*_*c*_ denote the number of variables in each spatial cluster and *C*_1_, …, *C*_*c*_ are the spatial cluster memberships. For a given threshold λ, the prediction strength *Pd* is defined as the median proportion of correctly allocated variables across all spatial clusters in the set-1.


(6) $$Pd\left(\lambda \right)={\mathrm{median}}_{j\in \{1,\;\ldots ,\;c\}}\frac{1}{m_{j}(m_{j}-1)}\sum_{i\neq i^{\prime }\in c_{j}}I\left[D_{\lambda }\left(i,\;i^{\prime }\right)=1\right]$$

This process is repeated for several random folds to determine the mean and standard error of the prediction strength. The optimal threshold is then chosen as the lowest value whose mean prediction strength is within one standard error of the maximum mean prediction strength over all possible thresholds.

### 2.4 SPA input for STOCSY

While SPA can identify spatial clusters, it cannot on its own identify the metabolites in a sample. To achieve this, we coupled SPA-identified spatial clusters with STOCSY. We first calculated each cluster’s intensity as a mean value of the local maxima peaks within the cluster and generated a new data matrix. We then input this data matrix into STOCSY. Because SPA groups variables that exhibit strong correlations, there is a chance that the correlations of highly concentrated metabolites at specific regions may overwhelm the metabolites present in low concentrations. Therefore, to ensure that Spatial Clustering Algorithm-Statistical Total Correlation Spectroscopy (SPA-STOCSY) captures all correlated clusters, we took advantage of a chemical reference such as DSS (4,4-dimethyl-4-s ilapentane-1-sulfonic acid), which we used as an internal standard. This is a complex compound having a singlet at 0.00 ppm. The distances between its peaks are relatively stable in all samples, so when each sample is aligned to this singlet at 0.00 ppm, all spectra are well aligned. When DSS is not used, other metabolites known to be present in the sample with high concentration could be used as a calibration reference. When choosing the alternate calibration reference, the chemicals should resonate at several frequencies along the spectrum and exhibit high correlations in SPA-STOCSY (Ulrich *et al.* 2008). Thus, the optimal value is the correlation threshold value when all DSS/reference metabolite resonances were captured by STOCSY. This value can change depending on the given sample and data, but we note that for all our samples it was 0.8. We then overlayed the clusters onto the spectrum and determined the corresponding resonances. For both known and unknown metabolites (those not annotated in the reference library), metabolite identification was possible using these distinguished signals and their resonances either by matching with a reference library or fragment-matching using the identified splitting pattern.

### 2.5 Identification of metabolites from SPA-STOCSY results

Using the SPA-STOCSY approach, we identified multiple groups of highly correlated clusters. These clusters, combined with peak picking, provide a collection of peaks that likely originate from the same metabolite. To aid the metabolite identification process, we generated an external reference library containing chemical shift information for over 200 metabolites.

To account for variations arising from different environmental factors, such as pH and temperature, we set a tolerance region of ±0.025 ppm for each metabolite cluster within the reference library. More specifically, when using SPA-STOCSY to map highly correlated clusters, the cluster is allowed the maximum step of ±0.025 ppm to match with candidates in the library. This approach helps maintain SPA-STOCSY’s robust performance in the face of internal and external factors that may affect chemical shift, such as the pH, sample composition, and temperature.

Then, for each metabolite within the reference library, we first noted its total number of clusters within the 0.5–4 ppm region. Similarly, we noted the number of metabolite-specific, highly correlated clusters detected by SPA-STOCSY analysis. We then divided the number of SPA-STOCSY-detected clusters by the total number of clusters from the reference library, resulting in a detection ratio for each metabolite. To ensure identification of metabolites with diverse complexities, we set a fixed detection threshold of 0.55. Only metabolites with a detection ratio surpassing this threshold were deemed as “identified metabolites.” This particular threshold value was determined by considering the number of clusters associated with different metabolites. For example, the threshold of 0.5 captures only half of the metabolite clusters from the reference library and is insufficient for metabolite identification. On the other hand, a threshold of 0.6 becomes too stringent, especially for metabolites with multiple clusters (for instance, a complex metabolite with nine clusters could still be considered identified if at least five clusters are detected). After careful consideration of all cases, we found that a detection ratio of 0.55 strikes a reasonable balance for most metabolites. It allows us to confidently claim metabolite identity by capturing a substantial portion of the metabolite’s clusters, while still accommodating the complexity and variability observed in the data.

However, applying a fixed detection ratio of 0.55 for metabolites with diverse complexities creates uncertainty for metabolites with a single cluster. That is, as STOCSY captures the dependencies among multiple clusters originating from the same metabolite, these are theoretically independent of other clusters in the case of single-cluster metabolites. Accordingly, we implemented a singlet-filter option in the detection step, meaning SPA-STOCSY will only keep single-cluster metabolites that are identified from the self-correlated group.

### 2.6 Statistical recoupling of variables (SRV)

To compare SPA performance with an existing spatial clustering method, we chose the statistical recoupling of variables (SRV) developed by Blaise *et al.* (2009). Let *X*_*n*×*p*_ denote the data matrix, with rows representing samples and columns representing spectral variables. Let us define


(7) $$L_{j}=\frac{\mathrm{covariance}}{\mathrm{correlation}}\left({x.}_{j},\;{x.}_{j+1}\right)=\sqrt{var\left({x.}_{j}\right)var\left({x.}_{j+1}\right)}=\sqrt{\frac{1}{n}\sum_{i=1}^{n}{\left(x_{i,j}-\bar{{x.}_{j}}\right)}^{2}\frac{1}{n}\sum_{i=1}^{n}{\left(x_{i,j+1}-\bar{{x.}_{j+1}}\right)}^{2}},$$

for *j* = 1, …, *p*. The boundaries of spectral clusters are identified by the local minima of the landscape function. The minimum number of variables in one cluster is determined by the resolution of the NMR spectra. Blaise *et al.* (2009) suggest discarding clusters with fewer than 10 variables. Representative intensities of spectral clusters are calculated as the mean of NMR signals assigned to each cluster. Super-clusters are then formed by aggregating clusters according to their correlation with neighboring ones. The authors suggest aggregating clusters with a correlation >0.9 and limiting the association to a maximum of three clusters to keep efficiency in areas of highly correlated signals.

### 2.7 NMR sampling

*Drosophila* head tissues (Elav-GAL4/w1118; +/+ strain; 12-day-old females, 40 heads per sample, *N* = 10) and human embryonic stem cells (hESCs; 1 million cells per sample; *N* = 22) were each dissolved in phosphate-buffered saline, pH 7.25, containing 10% D2O as a field frequency lock. One-dimensional ^1^H-NMR spectra were collected at 23°C using an 800 MHz NMR spectrometer (Bruker Corp.). DSS was used as the chemical shift internal standard for *Drosophila* tissues, while for hESCs we used 0.05 mM TSP (Trimethylsilyl propanoic acid). For *Drosophila* head tissues, for each free induction decay (FID), 128 transients were recorded at a repetition rate of 2 s per transient. To minimize the large water peak, the water signal was presaturated with a low power radio frequency pulse. To minimize the water peak for hESCs, the ZGESGP pulse sequence from the Bruker library was selected. The size of each FID was equal to 32K, the number of dummy scans was 4, and the spectral width was 12 ppm. 128 transients were recorded at a repetition rate of 1.7 s per transient.

Finally, spectra from both datasets were preprocessed in Topspin, including Fourier transform to the frequency domain, Gaussian filter, centering of the water peak to 4.7 ppm, binning, phasing, baseline correction, peak alignment to the internal standard, and normalization to the integral of the spectrum. To remove the dilution factor, we applied the probabilistic quotient normalization on the normalized dataset (Dieterle *et al.* 2006). Similar to others, we observed that the variables at baseline regions in the preprocessed data had notably high correlations. We estimated the baseline level by calculating five standard deviations from a noise region of the baseline and set the points with intensities less than the baseline level to zero, as suggested by Torgrip *et al.* (2008). Here, we considered the noise region 0.08–0.58 ppm.

### 2.8 Manual analysis of the NMR spectra by an NMR expert

We used Chenomx, Inc. software for traditional, manual analysis of the NMR spectra by NMR chemist who has extensive expertise using this software. To ensure rigorous analysis, the NMR chemist was blinded to the identity of the samples, performed all NMR data collection and preprocessing, and used the full spectral data for analysis because the software is designed for full spectral analysis. It typically takes four to five days to analyze one sample using Chenomx.

## 3 Results

### 3.1 SPA identifies strongly correlated functional units

In the NMR spectrum, each metabolite has unique signatures depending on its chemical and physical structure. As noted, the signature is represented by one or more structural units, which can be singlets, doublets, or multiplets in the spectra. SPA identifies these structural units even where many of the signals overlap (Fig. 2) because unlike existing clustering methods, which focus on individual peaks or the variation of peaks, SPA groups datapoints that belong to the same structural unit. It does so by finding contiguous groups of datapoints that exhibit stronger correlations since datapoints from the same multiplets correlate more strongly with each other than with those from other multiplets.

First, SPA determines the relationships among individual datapoints. After standard preprocessing, a typical NMR spectrum has more than 3000 datapoints that can generate about 5 000 000 pairs of correlations, each of which contains complex patterns. To calculate the correlation landscape, SPA first determines the window size, which is a type of bucketing. Bucketing is commonly used to control the peak shifts in NMR data, but the drawback is a loss of resolution (Forshed *et al.* 2003). We thus need high-resolution bucketing while keeping in mind that decreasing the size of the bucket can cause loss of spectral dependence (Blaise *et al.* 2009). Accordingly, SPA uses a PACF approach to determine the window size, thus balancing resolution quality and spectral dependence. To illustrate, assume a datapoint at location n and another data point at location n-k. The partial autocorrelation of these two points is the correlation of the two datapoints conditionally on the datapoints between them. This approach estimates a window size *k* where datapoints within this window have significantly stronger dependencies. To test the SPA’s performance, we first synthesized a spectrum using 10 random metabolites with complex spectra (Supplementary Fig. S1). After a series of calculations and filtering, the SPA produced spatial clusters that contained datapoints and peaks with the strongest correlation and without noise or baseline datapoints (Fig. 2b and c). Therefore, these clusters were more likely to be functional units, representing the structural information of the underlying metabolite.

### 3.2 SPA consistently outperforms SRV in discriminating signals from noise

**Figure 3.**
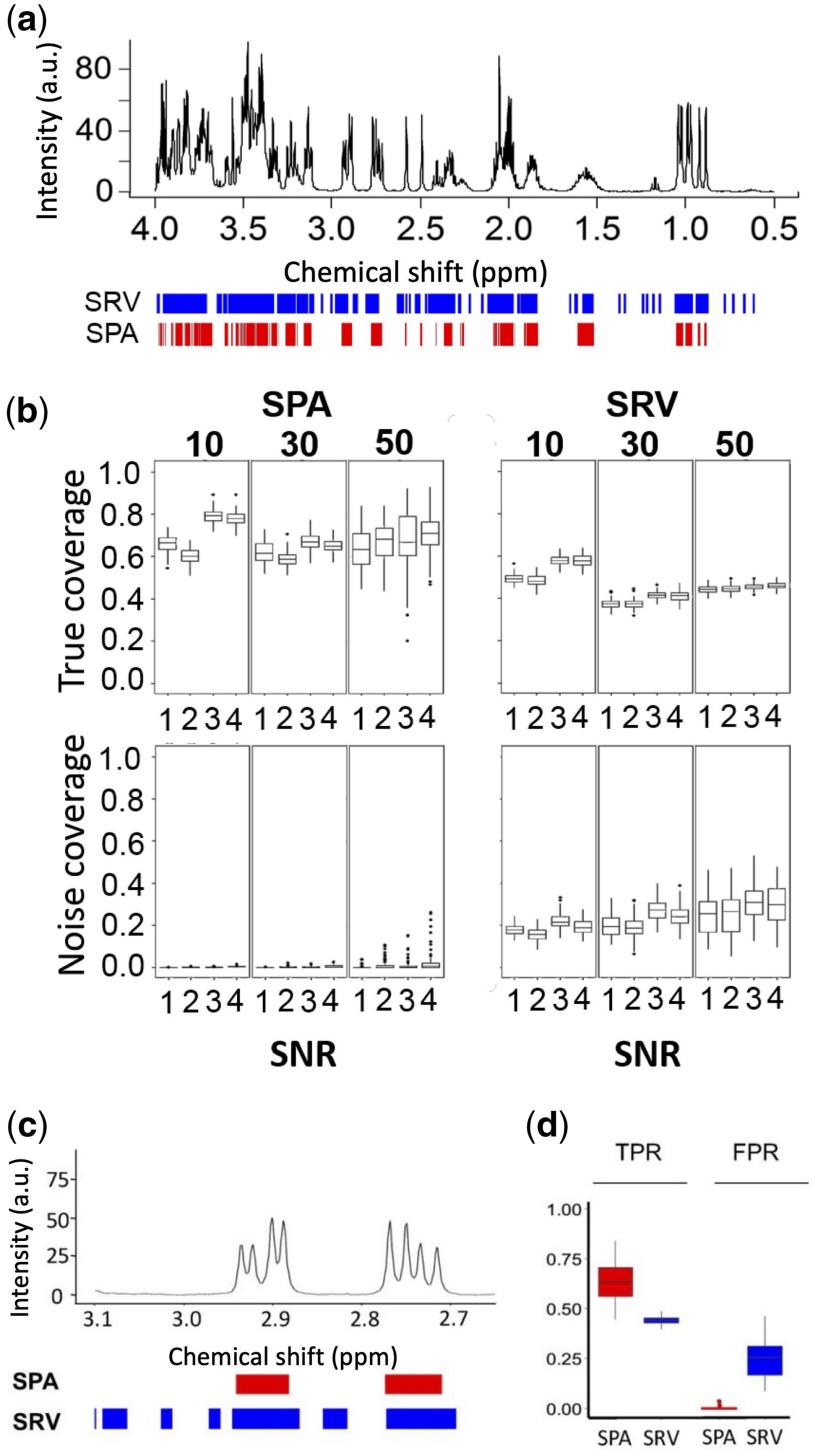
Comparison of the SPA and SRV clustering performance. (a) SPA (red) and SRV (blue) clustering on a simulated spectrum containing 10 metabolites. (b) The performance of each method was measured. *True signal coverage*: Proportion of detected true metabolite regions, measured as the percentage of the metabolite regions detected over the true metabolite resonance regions. *Noise coverage*: Proportion of detected regions in which no metabolite resonates. This is measured as the number of detected variables located in noise regions divided by the total number of noisy variables. Boxplots show the true signal coverage and noise coverage from simulated datasets using SRV and SPA on all the simulation scenarios (Supplementary Table S1). In each scenario, 100 simulations were conducted. (c) A synthesized spectrum containing 50 metabolites was analyzed by both methods. SPA discriminates between signals and noise better than SRV, as illustrated on a zoomed region of the spectrum. Boxes indicate assigned clusters (red: SPA; blue: SRV). (d) Boxplots show the true positive rate (TPR) and false positive rate (FPR) for SPA and SRV. SRV, Statistical Recoupling of Variables.

We then compared SPA to SRV (Blaise *et al.* 2009), another clustering method that calculates a landscape function as the ratio of covariance and correlation between neighboring spectral datapoints (Fig. 3a). We designed three sets of simulated spectra, in which the total number of metabolites per sample was fixed at 10, 30, or 50, respectively. We chose to increase the number of metabolites in the sample because the more metabolites, the greater number of overlapping regions where metabolites resonate. For each simulated set, we generated four scenarios by varying metabolites’ concentrations, sample size, and sample variations (Supplementary Table S1). With these different simulation parameters, each scenario had a different SNR (Supplementary Table S1). When analyzing the spectra, an ideal method would demonstrate high-true coverage (detection of true metabolite regions) and low-noise coverage (detection of regions in which there is no metabolite). For each scenario, we calculated SPA’s and SRV’s true coverage and noise coverage (Fig. 3b) and ran the analysis on 100 datasets (e.g. for a dataset with 50 samples, the total number of tested samples was 50 × 100). Increasing the total number of metabolites in the sample resulted in increased variability of both true and noise coverage for SPA, but only noise coverage for SRV. As expected, the true coverage increased with increased SNR for both methods, while the sample size did not affect the results (Fig. 3b). The differences between the SPA and SRV measurements were all significant according to the student’s *t*-test (Fig. 3c and d). SPA’s advantage over SRV was demonstrated by the clear difference in their respective true coverage and noise coverage ratios (Fig. 3). This finding is of critical importance because it indicates SPA reliably discriminates signals from noise.

### 3.3 SPA demonstrates a better balance between true coverage and noise coverage compared to traditional bucketing methods

The first step in traditional NMR data analysis is binning. One might reason that simple binning would be more effective than SPA in causing the spectral datapoints to cluster into metabolite signals and noise signals, so we assessed SPA’s performance against two traditional binning methods: standard binning and intelligent binning. Standard binning uniformly divides the NMR spectra into small bins of equal size using a fixed window size while intelligent binning allows flexibility in adapting the window size based on NMR peaks. Typically, however, each intelligent bin contains one NMR peak.

In our study, we generated 50 groups of simulated datasets. Within each group, we generated 50 NMR spectra, each containing 30 metabolites. Using each binning method independently, we split the NMR spectra into multiple buckets and then used peak picking to identify peaks within the spectra. The buckets containing peaks were considered metabolite regions, while the remaining buckets were classified as noise regions. We then calculated the true coverage and noise coverage for SPA and both binning methods combined with a peak-picking algorithm.

As shown in Supplementary Fig. S2, both binning methods heavily relied on the peak-picking methods to identify metabolite regions. Specifically, we adjusted the parameter of thresh.scale in the peak-picking method to achieve optimal results. We observed that decreasing the parameter thresh.scale led to an increase in both true-signal coverage and low-noise coverage. However, SPA demonstrated stability in achieving high true-signal coverage and low-noise coverage without the need for manual tuning of parameters, effectively retaining the most informative signals for subsequent steps. These results demonstrate SPA’s robustness and ability to automatically extract valuable information from NMR spectra, making it superior to traditional methods in metabolite region identification.

### 3.4 Coupling SPA and STOCSY achieves accurate identification of metabolites with both distinct and overlapping signals in the simulated data

While SPA can identify structural units in the spectra, it cannot by itself identify their corresponding metabolites. We therefore coupled the SPA with STOCSY, a method that uses statistical correlations between spectral data points, to identify signals originating from the same molecule (Cloarec *et al.* 2005). STOCSY has been used previously to explore the intra-and inter-metabolite correlations within raw NMR data, but high dimensionality and overlapping signals can make STOCSY readout and interpretation cumbersome (Cloarec *et al.* 2005). In SPA-STOCSY, however, we input the spatial clusters selected by SPA rather than raw NMR data. Therefore, the spectrum is interpreted as a series of spatial clusters rather than datapoints. SPA-STOCSY then provides correlations between these spatial clusters, allowing precise identification of corresponding metabolites.

**Figure 4.**
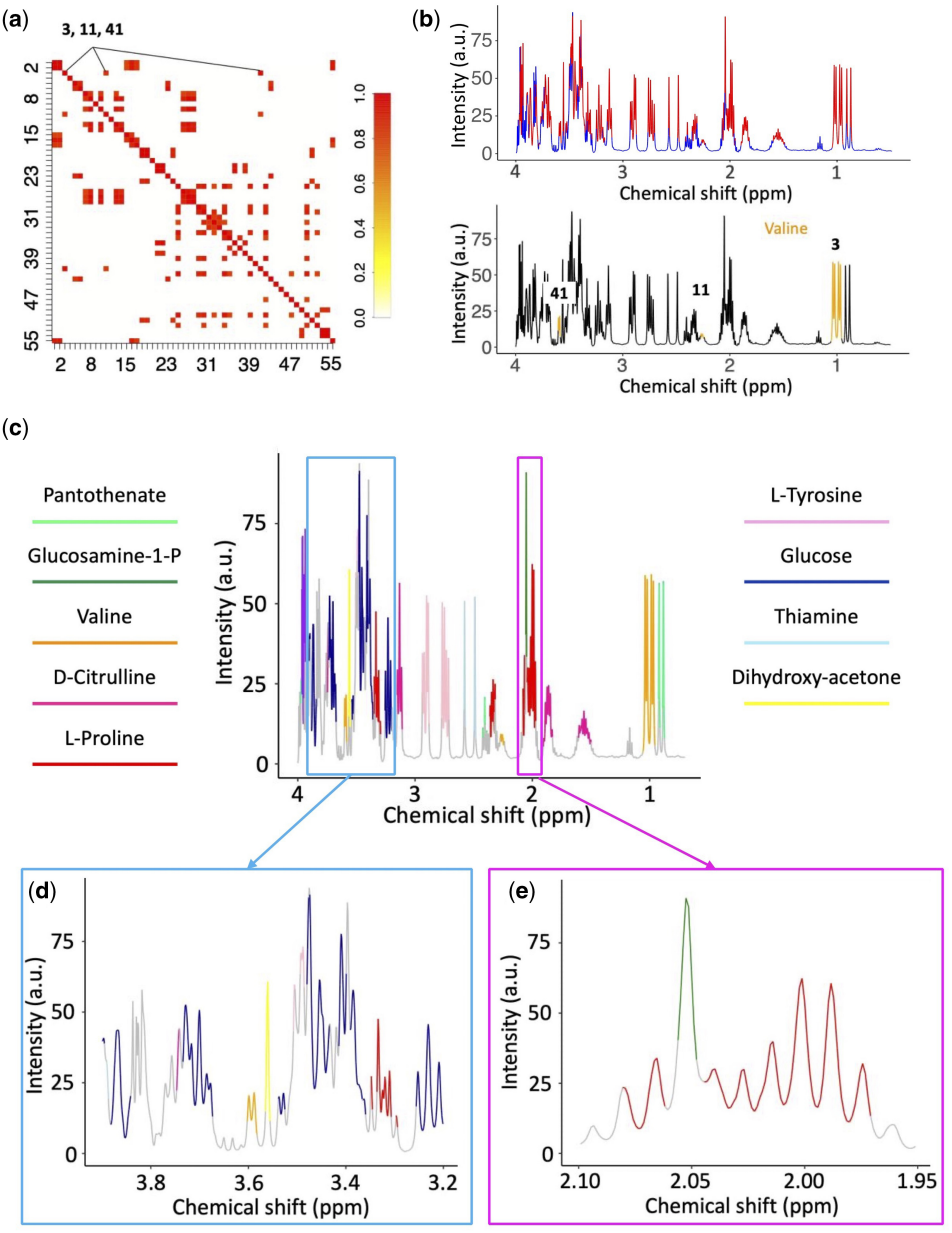
SPA coupled with STOCSY identifies connected fragments of molecules in the NMR spectra and thus, metabolites in the samples. (a) STOCSY is performed on spatial clusters obtained from the simulation dataset with 10 metabolites (Fig. 2a). Fifty-five spatial clusters are identified with a threshold of detection 0.8. (b) SPA clusters spectra into spectral regions (red) and gaps (blue). The example is given for clusters 3, 11, and 41, identified by SPA-STOCSY as highly correlated and therefore likely to belong to the same metabolite. Indeed, they belong to valine. (c) Nine out of 10 metabolites in the simulated dataset are correctly identified by SPA-STOCSY in a single pass. (d, e) Zoomed-in regions from (c). (d) SPA-STOCSY discriminates highly overlapping regions between 3.2 and 4.0 ppm, where the method identifies glucose, dihydroxy-acetone, l-tyrosine, and valine peaks. (e) SPA-STOCSY discriminates highly overlapping regions between 1.95 and 2.10 ppm, where the method identifies l-proline and glucosamine-1-phosphate (a singlet embedded in an unrelated multiplet).

We tested the SPA-STOCSY algorithm using simulated spectra with a set of 10 metabolites (spectrum shown in Fig. 2a) and a reference library of 50 metabolites. We first calculated each SPA-derived cluster’s intensity as a mean value of the peaks within the cluster and generated a new data matrix to be loaded into STOCSY. Using 0.8 as the threshold for SPA-STOCSY correlations, we identified the highly correlated clusters (Fig. 4a). Each set of STOCSY-derived highly correlated SPA clusters, when projected onto the mean spectrum, displayed the reconstructed signatures of the metabolites that might exist in the sample (Fig. 4b, upper panel). We then input spectrum resonances from the highly correlated clusters into the reference database to identify the metabolites. For example, valine resonated in three clusters, all of which were accurately identified by SPA-STOCSY (Fig. 4b, lower panel). Overall, SPA-STOCSY identified nine out of 10 metabolites present in the simulated dataset from the 50 metabolites in the reference database (Fig. 4c). Thus, we concluded that SPA-STOCSY-derived spatial clusters were close representations of the resonances from metabolites that existed in each sample. Moreover, STOCSY can correctly extract clusters that belong to the same molecule. To achieve this result, SPA must effectively capture the true metabolite regions (Fig. 3d) and STOCSY must accurately pick up these clusters (Fig. 4a).

Upon inspection of the selected metabolites, we recognized that some reconstructed signal regions were incomplete, such as the glucose signal regions at 3.2–4 ppm (Fig. 4d). This omission is caused by the overlapping signals from different molecules, which can reduce the existing correlations (Cloarec *et al.* 2005). However, as SPA-STOCSY identified the majority of glucose structural units, we were still able to identify this metabolite (Fig. 4d).

Peak overlapping is a universal challenge in NMR-based metabolomic profiling and many algorithms lose accuracy when overlapping spectral peaks are present in a sample. In contrast, SPA-STOCSY substantially aids the identification of metabolites because of its unique ability to identify overlapping peaks. In the SPA algorithm, the correlation landscape is sensitive to even minor changes in pair-wise correlations of the consecutive *k* datapoints, in which *k* is the window size. The fluctuation of the correlation landscape thus represents both the connectivity strength of high-correlation resonances that may originate from the same molecule and the separation capability of resonances from different molecules or overlapping peaks (Fig. 2b). Spatial clusters tend to split where overlapping occurs because this reduces the original strong correlations among datapoints representing the same metabolite multiplet. When we apply STOCSY, the split clusters describing the same functional unit can be recombined because they are highly correlated, thus reconstructing metabolite regions. We demonstrate this property in two examples: L-proline and glucosamine-1-phosphate N-acetyltransferase, which overlap at the 2.0 ppm region (Fig. 4e), and the glucose region between 3.2 and 4 ppm, where the method accurately identified glucose, dihydroxy-acetone, l-tyrosine, and valine peaks (Fig. 4d). These findings indicate that we can overcome the problem of overlap by correctly identifying and splitting the structural units in the overlapping regions. When correctly identified, structural units are coupled in the STOCSY algorithm and the true metabolite regions are reconstructed.

### 3.5 SPA-STOCSY provides automatic identification of metabolites

The SPA-STOCSY algorithm has several automated features that quickly and accurately identify candidate metabolites and potentially avoid human interference in processing NMR data. These automated features include (i) determining window size for the calculation of correlation landscape; (ii) deciding the threshold value for grouping datapoints that are likely from the same spatial cluster according to the correlation landscape; and (iii) identifying metabolites by matching highly correlated SPA clusters with the reference library. The first two parameters are determined automatically through exploration and investigation of the dataset under study. (Of note, we provide the user with an option to modify these parameters manually, if needed). To connect SPA-STOCSY data to the metabolite reference library, we designed an algorithm that provides a readout of all candidate molecules by matching spatial clusters resonances with the reference resonances. Specifically, we first identify each spatial cluster’s peaks using the local maximum for peak detection. Highly correlated spatial clusters can then be transformed into a series of highly correlated peaks, which the algorithm inputs into the reference library to find corresponding metabolites.

### 3.6 Identification of metabolites in biological samples

**Figure 5.**
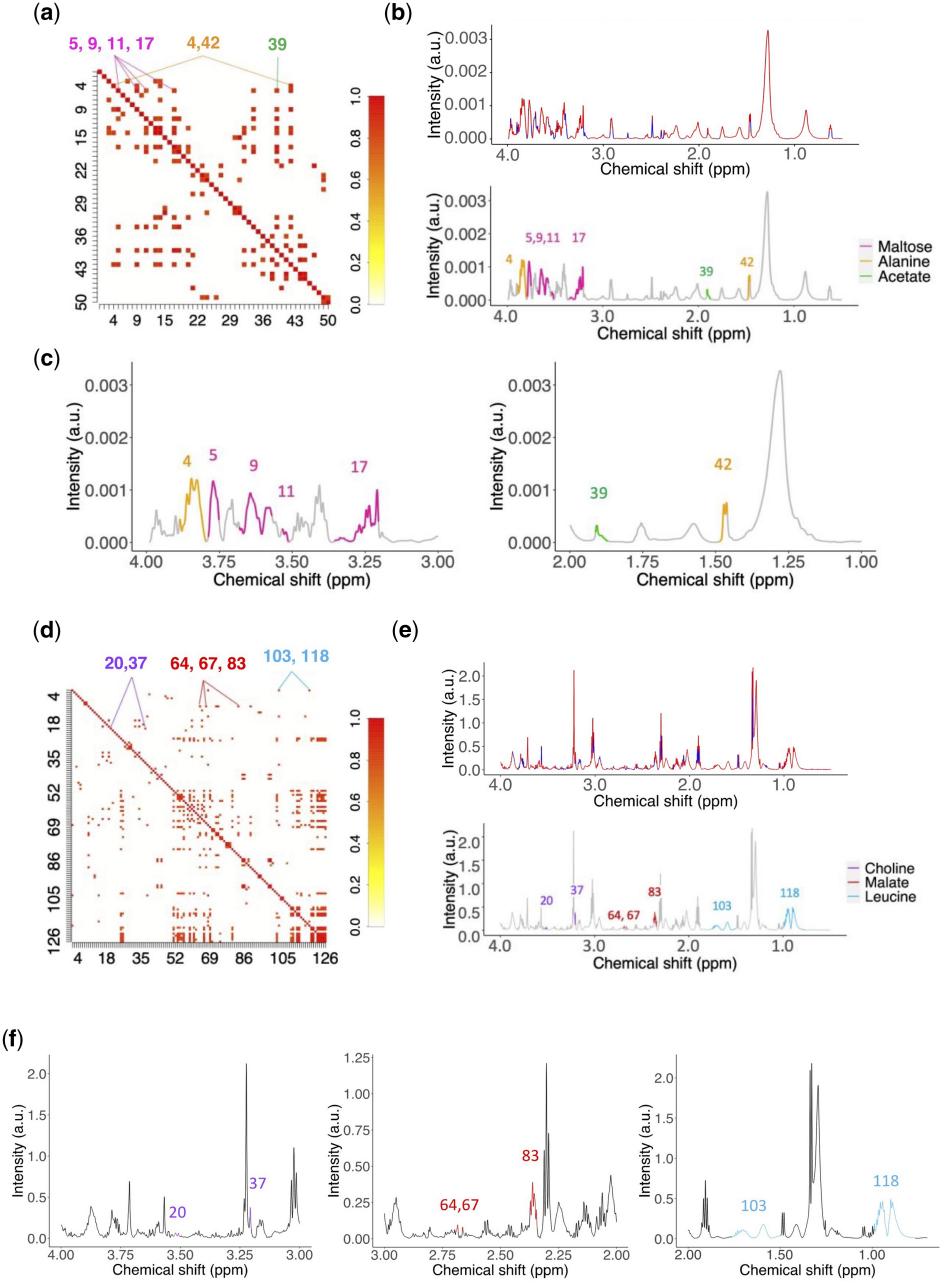
SPA-STOCSY identifies metabolites in the *Drosophila melanogaster* tissue and human embryonic stem cells (hESCs). (a) SPA-STOCSY identifies 50 highly correlated clusters at a detection threshold of 0.8 in the *Drosophila* data. (b) SPA clusters the mean spectrum (*N* = 10) into structural units. The spectrum is color-coded by the correlation landscape values (red: strong correlations; blue: weak correlations). Clusters with high correlations are deconstructed into original resonance frequencies and the identities of the corresponding metabolites are obtained. Maltose, alanine, and acetate are given as examples. (c) Amplified version for the visualization of highly correlated clusters and the corresponding metabolites from (b). (d) SPA-STOCSY identifies 126 highly correlated clusters in hESC data. (e) SPA clusters the mean spectrum (*N* = 22) into structural units. The spectrum is color-coded by the correlation landscape values (red: strong correlations; blue: weak correlations). Clusters with high correlations are deconstructed into original resonance frequencies and the identities of the corresponding metabolites are obtained. Choline, malate, and leucine are given as examples. (f) Amplified version for the visualization of highly correlated clusters and the corresponding metabolites from (e). SPA-STOCSY accurately identifies molecules with complex signatures regardless of the overlapping and/or splitting regions.

We tested SPA-STOCSY’s performance on biological samples, using *ex vivo* tissues from *Drosophila melanogaster* and cultured human embryonic stem cells (hESCs) to generate diverse datasets. In *Drosophila melanogaster* head homogenates containing mostly brain mass, we determined that the optimal window size for scanning the correlation landscape using PACF was seven and we used the tricube kernel smoother to calculate the correlation landscape. Following application of SPA, we observed that high correlation landscape values were always localized to signal regions and we identified 50 clusters that exhibited strong correlations (Fig. 5a). In addition, some regions had lower correlation landscape values and spatial clusters were split there. The sharp decline of correlation landscape values indicated overlap; the splitting was more common in regions between 3 and 4 ppm, abundant in overlapping resonances from different metabolites. We then input the data into the STOCSY part of the algorithm. Here, the most important step was to determine the detection threshold, i.e. the optimal correlation that provides the maximum number of meaningful variables. As described in Section 2.4, the optimal correlation threshold for the *Drosophila* head NMR dataset was calculated as 0.8 based on the DDS resonance clusters.

We set the automatic detection ratio at 0.55 (see Section 2.5) and identified 131 metabolites (Supplementary Table S2; some examples are presented in Fig. 5a–c). As shown, the complexity of the resonances clustered by SPA-STOCSY ranged from 1 (acetate) to >5 (maltose), including the regions of highly overlapping signals, such as between 3.00 and 4.00 ppm. An expert NMR chemist also performed an analysis of the same samples using commercially available software, Chenomx (Chenomx Inc.). This analysis identified 81 metabolites (three of which were not in our library). To discern the differences, we visually inspected the identified metabolites, focusing on those that resonated from 0.00 to 4.00 ppm. SPA-STOCSY and Chenomx identified 55 common metabolites. There are several reasons for their differing performance: Chenomx sets the baseline automatically and it under-samples because it considers concentration of each metabolite within a given peak, while SPA-STOCSY over-samples because it captures metabolites based on peak detection and cluster positions only, without taking into account metabolite concentrations.

Using a similar approach, we next applied SPA-STOCSY to 22 NMR spectra from hESCs. Using PACF, the window size was set at 11. The SPA generated 126 clusters in total. Due to technical difficulties during sample collection, the TSP resonances were distorted. So, to determine the optimal correlation threshold for this set of spectra, we took the two clusters from choline as the reference and calculated this threshold at 0.8. When we set the detection ratio at 0.55, SPA-STOCSY identified 126 clusters and 87 metabolites in the spectra. Three representative metabolites are shown in Fig. 5d–f. Despite the higher complexity of the hESC NMR data compared to the *Drosophila* samples, SPA-STOCSY still captured highly correlated clusters from the same metabolite in diversified regions, with clusters ranging from singlets to multiplets. For most of them, SPA-STOCSY achieved a detection ratio of at least 0.5. Chenomx profiling of the same hESCs data by an NMR expert identified 24 metabolites (Supplementary Table S3). Compared to manual profiling, SPA-STOCSY is more efficient and offers more testable candidates. We thus believe that SPA-STOCSY is the first method to enable automated analysis of spectra with unknown number and type of metabolites with precision and speed (7 min).

## 4 Discussion

In this study, we introduce a new algorithm, SPA-STOCSY, as an untargeted NMR metabolome profiling tool. The SPA part of the algorithm greatly reduces the high dimensionality of the NMR dataset by transforming the datapoints into spatial clusters that represent functional units. SPA groups adjacent variables that potentially originate from the same functional unit and groups different variables according to their average correlations, which can capture small changes in pair-wise correlations among variables along the spectrum within a determined window size *k*. Spatial clusters split where a sudden decline in average correlations occurs, as in the case of overlapping resonances. Therefore, SPA aids the identification of metabolite regions by detecting overlapping components.

When analyzed by an NMR expert, noise and overlapping peaks are the major factors that can introduce bias in metabolite identification. SPA, on the other hand, makes full use of the spectral information to limit the influence of subjective bias. SPA outperforms SRV in successfully separating signal regions from noise regions. This clear signal-to-noise separation provides a good start for an expert’s analysis and decreases bias. SPA delivers a noise-free spectrum within 2 min, which can ease preprocessing without losing marginal spectral information.

SPA boosts its performance when coupled with STOCSY. Several of SPA’s significant features enable accurate metabolite detection, as shown in both our simulation studies and biological samples. For metabolite identification, a crucial task is separating the overlapping peaks. SPA itself can only separate signals from noise, but not metabolite from metabolite. And for NMR experts, these separation boundaries are mostly decided based on their experience, raising the problem of subjectivity and information loss if they only focus on the dominant peaks. SPA-STOCSY can set these separation boundaries with less ambiguity by applying the intrinsic features of concentration heterogeneity among metabolites within the sample. These features can help SPA-STOCSY separate small peaks from the dominant group if they are not from the same metabolites and offer a detailed annotation for the high-overlapping regions.

Further, biological samples are complex and their composition and total number of metabolites are unknown. One metabolite may be present in some of the samples, but not in others. Despite this complexity, the connectivity of the underlying signals from the same metabolite persists and can be captured. SPA-STOCSY uses covariance patterns from a dataset to recover such connectivity, thereby identifying a given sample’s missing signals that are present in other samples in the same dataset.

With these features, SPA-STOCSY outperforms current automated identification and visualization methods. For example, MetaboID requires the user to select candidate metabolites, which introduces operator bias (Mackinnon *et al.* 2013). MetaboHunter enables automatic identification of metabolites (Tulpan *et al.* 2011), but it matches individual peaks to the reference library rather than a group of peaks that originate from the same structural unit, resulting in a high false detection rate. MetaboHunter’s mutual exclusion of peaks also neglects overlapping signals, increasing the need to visually inspect the spectra for the metabolite readouts. BAYESIL segments spectra into small blocks and uses the probabilistic graphical model to estimate the metabolite concentrations and chemical shifts. Thus, BAYESIL is more suited for specific biofluids with sub-libraries curated from serum, plasma, or cerebrospinal fluid (Ravanbakhsh *et al.* 2015). FOCUS is an integrated tool that identifies metabolites based on peak picking and matching, with the peaks extracted in pure compounds’ reference spectra (Alonso *et al.* 2014). Although FOCUS’s matching score considers peak correlations with peak overlapping, it is undermined by a high proportion of zero-intensity peaks. ASICS is a linear-based tool whose accuracy depends heavily on a dedicated shifting algorithm that contains two steps: global shift and local peak distortions (Tardivel *et al.* 2017, Lefort *et al.* 2019). In the biological samples, the maximally allowed global shift needs to be well-tuned for the best alignment. This is a time-consuming task because samples are intrinsically heterogeneous and different metabolites have different sensitivities to shift adjustments. Thus, it is hard to set a standard for all of them and it is inconvenient to tune each separately. Consequently, while using residuals to find the best peak distortions, ASICS is likely to align reference spectra to the wrong clusters in heavily overlapping regions.

Despite the good performance of SPA-STOCSY compared to these other methods and manual analysis, we recognize potential limitations and have included some extra steps to address these intrinsic concerns. First, SPA-STOCSY depends on the homogeneity of the investigated samples (Alves *et al.* 2009). This suggests that the samples selected to explore structural connectivity should be similar in composition and the peak locations should be well aligned. Specifically, we assume that the correlation pattern of the dataset directly reflects the strength of proton connectivity. If the spectra in the dataset are not well aligned or the phase is not properly corrected, the correlation pattern may not completely represent the structural information of metabolites in the sample. To address this concern, we used a chemical reference, DSS, as an internal standard. Although there is a chance that small positional variation may exist in the spectra, the SPA grouping mechanism can overcome it by proper determination of the window size *k* for the calculation of correlation landscape. As we use average correlation instead of point-to-point correlation, a tolerance of minor perturbation is embedded. Secondly, SPA captures signal variation and differentiates them from noise based on the assumption that the true concentrations of underlying metabolites are not identical across samples. This concern is easily solved with the intrinsic heterogeneity in biological samples, as no pair is identical.

Further, to accommodate possible shifting due to different sample pH, ion strength, temperature, etc., we allow a chemical shift of ±0.025 ppm when matching SPA-STOCSY and the reference library. Finally, by using a fixed detection ratio to assess the presence of metabolites with varying complexities, the metabolites with multiple clusters have higher identification confidence compared to single-cluster metabolites. To address the uncertainty associated with single-cluster metabolites, we introduced a singlet filter during the identification process. Leveraging this assumption, we limited the identification of single-cluster metabolites to those clusters that were self-correlated only in the STOCSY results. By doing so, we reduced the uncertainty and minimized false positives in the identification results.

In this work, we did not attempt to directly compare SPA-STOCSY’s performance to the widely used, operator-based Chenomx analysis as this would require extensive experimental validation to ascertain each detected metabolite in the sample. Moreover, both methods have their advantages and disadvantages. Overall, SPA-STOCSY performs comparably to Chenomx analysis in identifying candidate metabolites, but it eliminates the possibility of operator bias while returning the results in only 7 min of computation time. It also outperforms SRV by capturing a higher percentage of the signal regions and the close-to-zero noise regions. Furthermore, because it summarizes the results from NMR spectra directly, SPA-STOCSY takes the library as a reference but is not limited to the specific library. Finally, unlike previous identification methods (Hao *et al.* 2012, Ravanbakhsh *et al.* 2015, Lefort *et al.* 2019), SPA- STOCSY identifies metabolites that are not annotated in the library by highlighting the correlated functional units with no match to any annotated metabolite. Accordingly, SPA-STOCSY offers new insights about metabolite compositions in diversified systems and offers researchers and clinicians a fast, highly accurate, and unbiased tool for NMR analysis, with several key advantages over existing methods.

## Supplementary Material

btad593_Supplementary_Data
