# Supplementary material for: SPA-STOCSY: an automated tool for identifying annotated and non-annotated metabolites in high-throughput NMR spectra
Source: Bioinformatics. 2023 Oct 4;39(10):btad593. doi: 10.1093/bioinformatics/btad593 (PMC10568371; doi:10.1093/bioinformatics/btad593)
Supplement: btad593_Supplementary_Data [file btad593_supplementary_data.zip › supplementary_table2.pdf]

Supplementary Table 2 | Metabolite identification in *Drosophila* data using Chenomx and SPA-STOCSY

Candidate metabolites	SPA-STOCSY identified	Number of peaks (0 ppm to 4 ppm)	Number of peaks (4 ppm to 10 ppm)	Chenomx profiling	Comments
1,3-Dihydroxyacetone	Y	1	1	Y	
1,3-Dimethylurate	Y	2	0	N	Overlapping
1,6-Anhydro-beta-D-Glucose	Y	4	3	Y	
2-Ethylacrylate	N	2	2	Y	
2-Hydroxyphenylacetate	Y	1	4	N	1 peak between 0-4 (singlet)
2-Hydroxyvalerate	Y	5	1	N	Not fit
2-Methylglutarate	Y	5	0	N	Not fit
2-Octenoate	Y	5	2	N	Overlapping
2-Oxocaproate	Y	3	0	N	Not fit
2-Oxoglutarate	Y	2	0	Y	
2-Phosphoglycerate	Y	2	1	Y	
3-Chlorotyrosine	Y	3	3	N	Overlapping
3-Hydromuconate	Y	1	1	N	1 peak between 0-4 (doublet)
3-Hydroxy-3-methylglutarate	Y	3	0	N	Overlapping
3-Hydroxybutyrate	Y	3	1	N	Not fit
3-Hydroxyisovalerate	Y	2	0	N	Overlapping
3-Hydroxykynurenine	Y	1	4	N	1 peak between 0-4 (doublet)
3-Hydroxyphenylacetate	Y	1	4	N	1 peak between 0-4 (singlet)
3-Methylxanthine	Y	1	1	N	1 peak between 0-4 (singlet)
4-Aminobutyrate	Y	3	0	Y	
4-Aminohippurate	Y	1	3	N	1 peak between 0-4 (doublet)
4-Hydroxy-3-methoxymandelate	Y	1	4	Y	
4-Hydroxyphenylacetate	Y	1	2	N	1 peak between 0-4 (singlet)
4-Pyridoxate	Y	1	2	N	1 peak between 0-4 (singlet)
5-Aminolevulinate	Y	4	1	N	Not fit
5-Hydroxyindole-3-acetate	Y	1	4	N	1 peak between 0-4 (singlet)
5-Hydroxytryptophan	Y	2	6	N	Overlapping
5-Methoxysalicylate	Y	1	3	N	1 peak between 0-4 (singlet)
Acetamide	Y	1	2	Y	
Acetaminophen	Y	1	2	N	1 peak between 0-4 (singlet)
Acetate	Y	1	0	Y	
Acetoacetate	N	2	0	Y	
Acetone	Y	1	0	Y	
Acetylsalicylate	Y	1	4	N	1 peak between 0-4 (singlet)
Adenine	N	0	2	Y	
Adenosine	Y	2	6	Y	
Alanine	Y	2	0	Y	
AMP	N	0	8	Y	
Anserine	Y	7	4	N	Not fit
Arabinitol	Y	7	0	Y	
Arabinose	Y	15	9	N	Overlapping
Arginine	Y	7	2	Y	
Ascorbate	N	2	2	Y	
Asparagine	Y	3	2	N	Not fit
Aspartate	Y	3	0	Y	
beta-Alanine	N	2	0	Y	
Butyrate	Y	3	0	N	Not fit
Cadaverine	Y	3	0	N	Overlapping
Caffeine	Y	3	1	N	Overlapping

Caprate	Y	9	0	N	Overlapping
Caprylate	Y	7	0	N	Overlapping
Carnitine	Y	5	1	N	Overlapping
Carnosine	Y	6	4	N	Overlapping
Choline	Y	2	1	Y	
Cis-aconitate	Y	1	1	Y	
Citrate	N	2	0	Y	
Citrulline	Y	7	1	N	Not fit
Creatinine	Y	1	1	Y	
Cysteine	Y	3	0	Y	
Dimethyl sulfone	Y	1	0	Y	
Dimethylamine	Y	1	0	Y	
DSS	Y	4	0	Y	
dTTP	Y	3	6	N	Not fit
Ethanol	N	2	0	Y	
Ethanolamine	N	2	0	Y	
Ethylene glycol	Y	1	0	Y	
Ferulate	Y	1	5	N	1 peak between 0-4 (singlet)
Fructose	Y	11	3	N	Overlapping
Fumarate	N	0	1	Y	
Galactarate	Y	1	1	Y	
Galactitol	Y	3	0	Y	
Galactonate	N	5	1	Y	
Glucarate	Y	1	3	N	1 peak between 0-4 (triplet)
Glucitol	Y	8	0	Y	
Gluconate	Y	4	2	N	Overlapping
Glucose	Y	12	2	Y	
Glutamate	Y	5	0	Y	
Glutamine	N	5	2	Y	
Glutaric acid monomethyl ester	Y	4	0	N	Not fit
Glycerate	N	2	1	Y	
Glycerol	Y	3	0	Y	
Glycine	Y	1	0	Y	
Glycolate	Y	1	0	Y	
Guanidoacetate	Y	1	0	Y	
Guanosine	Y	2	6	N	Not fit
Hippurate	Y	1	6	N	1 peak between 0-4 (doublet)
Histamine	Y	2	2	Y	
Histidine	Y	3	2	Y	
Homogentisate	Y	1	2	N	1 peak between 0-4 (singlet)
Homoserine	Y	2	0	Y	
Homovanillate	Y	2	3	N	Overlapping
Indole-3-acetate	Y	1	6	N	1 peak between 0-4 (singlet)
Inosine	Y	2	6	Y	
Isocitrate	Y	3	1	Y	
Isoleucine	N	6	0	Y	
Isopropanol	Y	1	1	N	Not fit
Isovalerate	Y	3	0	N	Not fit
Kynurenine	Y	1	5	N	1 peak between 0-4 (doublet)
lactate	N	1	1	Y	
Lactose	Y	24	4	N	Overlapping
Leucine	N	6	0	Y	
Lysine	N	7	0	Y	
Malate	N	2	1	Y	

Malonate	Y	1	0	Y	
Maltose	Y	24	4	Y	
Mannitol	Y	4	0	Y	
Mannose	Y	12	2	N	Overlapping
Methanol	Y	1	0	Y	
Methionine	N	5	0	Y	
Methylamine	Y	1	0	N	1 peak between 0-4 (singlet)
Myo-inositol	Y	3	1	N	Overlapping
N-Acetylaspartate	N	3	2	Y	
N-Acetylcysteine	Y	3	2	Y	
N-Acetylglutamate	Y	4	2	N	Not fit
N-Acetylglutamine	Y	5	4	Y	
N-Acetylserotonin	Y	3	6	N	Overlapping
N-Acetyltyrosine	Y	3	4	Y	
NADH	Y	2	17	N	Overlapping
Nicotinurate	Y	1	5	N	1 peak between 0-4 (singlet)
N-Methylhydantoin	Y	1	1	Y	
O-Phosphocholine	Y	2	1	Y	
O-Phosphoethanolamine	N	2	0	Y	
O-Phosphoserine	Y	1	3	N	1 peak between 0-4 (quadruplets)
Ornithine	Y	5	0	N	Not fit
Pantothenate	Y	8	1	N	Not fit
p-Cresol	Y	1	2	N	1 peak between 0-4 (singlet)
Phenylacetate	Y	1	3	N	1 peak between 0-4 (singlet)
Phenylalanine	Y	3	3	N	Overlapping
Proline	N	6	1	Y	
Pyridoxine	Y	1	3	Y	
Pyruvate	Y	1	0	Y	
Ribose	Y	15	9	N	Overlapping
Salicylurate	Y	1	5	N	1 peak between 0-4 (singlet)
Sarcosine	N	2	0	Y	
Serine	Y	3	0	Y	
Serotonin	Y	2	5	N	Overlapping
sn-Glycero-3-phosphocholine	Y	7	1	Y	
S-Sulfocysteine	N	2	1	Y	
succinate	Y	1	0	Y	
Sucrose	Y	11	3	N	Overlapping
Taurine	Y	2	0	Y	
Threonate	Y	3	1	Y	
Threonine	N	2	1	Y	
Theophylline	Y	2	1	N	Overlapping
Thymine	Y	1	1	N	1 peak between 0-4 (singlet)
Thymol	Y	3	3	Y	
trans-4-Hydroxy-L-proline	Y	4	2	N	Overlapping
trans-Aconitate	Y	1	1	N	1 peak between 0-4 (singlet)
Trimethylamine	Y	1	0	Y	
Trimethylamine N-oxide	Y	1	0	Y	
Tropate	Y	2	4	N	Overlapping
Tryptophan	Y	2	7	N	Overlapping
Tyrosine	Y	3	2	N	Overlapping
UDP-glucuronate	Y	3	10	N	Not fit
Valine	N	4	0	Y	
Vanillate	Y	1	3	N	1 peak between 0-4 (singlet)
Xylose	Y	10	2	N	Overlapping
