# Supplementary material for: SPA-STOCSY: an automated tool for identifying annotated and non-annotated metabolites in high-throughput NMR spectra
Source: Bioinformatics. 2023 Oct 4;39(10):btad593. doi: 10.1093/bioinformatics/btad593 (PMC10568371; doi:10.1093/bioinformatics/btad593)
Supplement: btad593_Supplementary_Data [file btad593_supplementary_data.zip › Han Wang Supplementary_data FINAL.pdf]

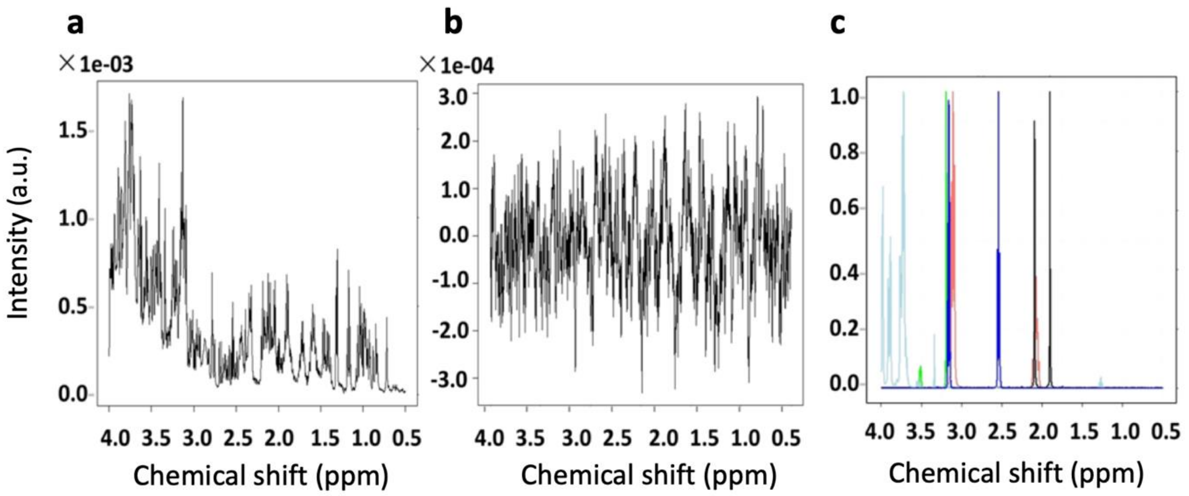

Supplementary Fig. 1 | NMR simulation model. a, A simulated spectrum with 50 metabolites. **b**, Simulated noise spectrum. **c**, A reference spectrum generated from five pure compound spectra downloaded from the BMRB database. The reference library data were Fourier Transformed, truncated to 0.5 to 4ppm region, and scaled to their highest peak. The intensity of the peaks is directly proportional to the metabolite concentration. Scaling enables manipulation of the pseudo concentration of a given metabolite in the simulation model.

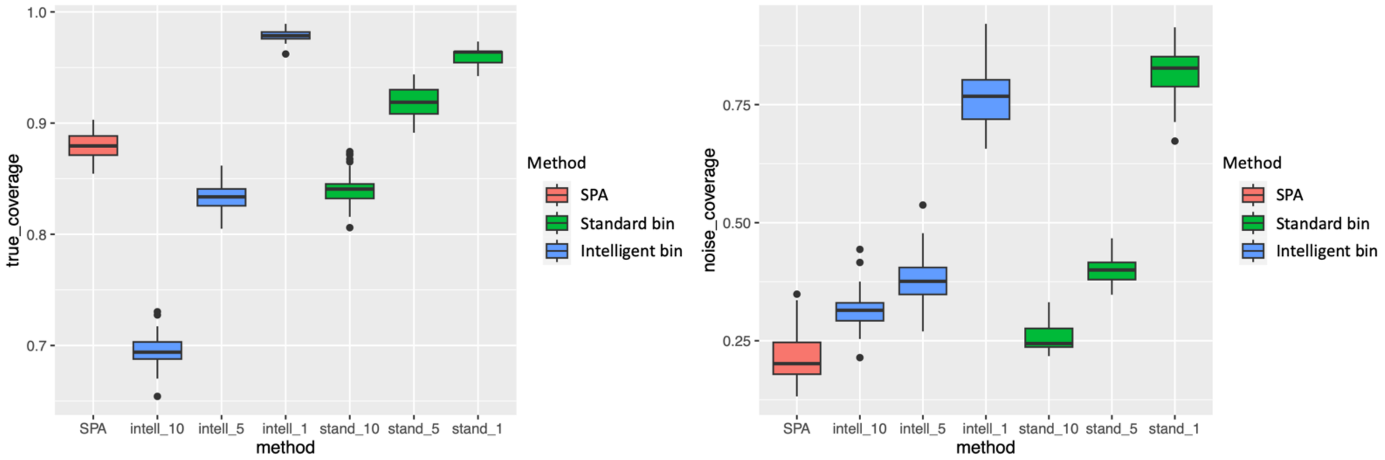

Supplementary Fig. 2 | True-signal coverage and noise coverage comparison between SPA, standard, and intelligent binning. Binning methods are implemented together with the peak picking algorithm. The parameter thresh.scale in the peak picking algorithm is set to 1, 5, or 10 to show the performance differences. The clustering performance for different methods and parameters was assessed by true-signal coverage and noise coverage.

Parameters / Scenarios	γ	ϕ	n	SNR_10	SNR_30	SNR_50
1	60	12	100	26.4	79.2	132
2	60	12	50	52.8	158.4	264
3	60	25	100	74.5	223.5	372.5
4	60	25	50	149	447	745

Supplementary Table 1 | Three sets of simulated datasets were generated containing 10, 30, or 50 metabolites. In each set, four scenarios were designed with different simulation parameters. ***Gamma,*** γ denotes the average concentration of metabolites in the sample; ***Phi,*** φ denotes the variance of different metabolite concentrations in the sample; ***n*** denotes the sample size. The signal-to-noise ratio (SNR) is calculated as the ratio of the variance of signals and the variance of the noise. With different simulation parameters, the SNR varies between each scenario and each set.
