# Supplementary material for: SPA-STOCSY: an automated tool for identifying annotated and non-annotated metabolites in high-throughput NMR spectra
Source: Bioinformatics. 2023 Oct 4;39(10):btad593. doi: 10.1093/bioinformatics/btad593 (PMC10568371; doi:10.1093/bioinformatics/btad593)
Supplement: btad593_Supplementary_Data [file btad593_supplementary_data.zip › Supplementary Table 3 FINAL.pdf]

Supplementary Table 3 | Metabolites identifiedin hESC NMR data using Chenomx. SPA-STOCSY detection data for the same metabolites are presented.

Metabolites identified with Chenomx	Total clusters	Number of clusters (0ppm to 4ppm)	Number of clusters identified	Detection ratio
4-Aminobutyrate	3	3	3	1
Myo-inositol	4	3	3	1
Choline	3	2	2	1
O-Phosphocholine	3	2	2	1
Glycine	1	1	1	1
Lactate	2	1	1	1
Nicotinurate	6	1	1	1
Pyruvate	1	1	1	1
Leucine	6	6	5	0.833
Tyrosine	5	3	2	0.667
Proline	7	6	3	0.5
UDP-galactose	15	4	2	0.5
Valine	4	4	2	0.5
Alanine	2	2	1	0.5
Glutamate	5	5	2	0.4
Glutamine	7	5	2	0.4
Isoleucine	6	6	2	0.333
Phenylalanine	6	3	1	0.333
sn-Glycero-3-phosphocholine	8	7	2	0.286
DSS-d6 (Chemical Shape Indicator)	1	0	0	no peak in 0ppm - 4 ppm
Glucose-6-phosphate	10	8	0	not in library
AMP	8	2	0	not in library
UMP	5	1	0	not in library
Fumarate	1	0	0	not in library
